# Tailoring parameter distributions to specific germplasm: impact on crop model-based ideotyping

**DOI:** 10.1038/s41598-019-54810-x

**Published:** 2019-12-04

**Authors:** Livia Paleari, Ermes Movedi, Fosco Mattia Vesely, Roberto Confalonieri

**Affiliations:** University of Milan, ESP, Cassandra lab, via Celoria 2, 20133 Milan, Italy

**Keywords:** Plant sciences, Climate sciences

## Abstract

Crop models are increasingly used to identify promising ideotypes for given environmental and management conditions. However, uncertainty must be properly managed to maximize the *in vivo* realizability of ideotypes. We focused on the impact of adopting germplasm-specific distributions while exploring potential combinations of traits. A field experiment was conducted on 43 Italian rice varieties representative of the Italian rice germplasm, where the following traits were measured: light extinction coefficient, radiation use efficiency, specific leaf area at emergence and tillering. Data were used to derive germplasm-specific distributions, which were used to re-run a previous modelling experiment aimed at identifying optimal combinations of plant trait values. The analysis, performed using the rice model WARM and sensitivity analysis techniques, was conducted under current conditions and climate change scenarios. Results revealed that the adoption of germplasm-specific distributions may markedly affect ideotyping, especially for the identification of most promising traits. A re-ranking of some of the most relevant parameters was observed (radiation use efficiency shifted from 4th to 1st), without clear relationships between changes in rankings and differences in distributions for single traits. Ideotype profiles (i.e., values of the ideotype traits) were instead more consistent, although differences in trait values were found.

## Introduction

Since the introduction of the “selection for ideotype” concept by Donald in 1968^1^ as an alternative approach to “selection for yield” or “defect elimination”, the definition of the optimal combination of plant traits for a given environment and a specific purpose (“ideotyping”) has increasingly catalysed the attention of the breeders’ community. Ideotypes have been defined for a variety of species (e.g., grain cereals^2–4^, legumes^5,6^, and fruit trees^7,8^) and customized to account for specific objective functions (e.g., increasing resources use efficiency^9^, product quality^10^, or alleviating negative impacts of climate change^2,4^).

In this context, the potential of crop models to explicitly account for genotype (G) × environment (E) × management (M) interactions has widely encouraged their use to support ideotype design. Perturbation of model parameters corresponding to plant traits allows indeed quantifying the impact of varying one or more plant features on whole-crop performance^11^ in response to heterogeneous spatial^12^ and temporal^2^ conditions. This allows deriving ideotypes specific for different environmental and management contexts, ultimately to drive the development of new, well-adapted varieties^13^.

A key step in model-aided ideotype design is defining the extent of parameters perturbation, which should not exceed the actual genetic variability of the corresponding trait^14^. This is a critical point regardless of the methodology used to conduct the ideotyping study, which can be based on global sensitivity analysis^5,15–17^, grid search^18^, parameter perturbation at specific values^19^, or on optimization tools^7,10^. A common way to deal with this issue is to apply plausible variations to a default parameter set^20^ or to the parameterization defined for a reference variety^21^. Alternative approaches, aimed at increasing ideotype feasibility, explore hypotheses of trait improvement by accounting for statistical distribution of model parameters^4^ and, when available, also for correlations among traits^8^. The underlying assumption is that the probability density of a trait is representative of its dispersion and possibility of selection, with, e.g., higher frequency for values close to the mean in case the distribution is normal^8^.

However, observations used to derive ranges/distributions for parameters perturbation are rarely representative of a specific pool of genotypes; rather they provide an indication of the variability available within the species, by assuming the sample of genotypes on which observations were collected as representative. This may lead to ideotypes that are hardly achievable with the genetic resources actually available within a specific breeding program.

Genetic improvement is tightly linked to the actual variability available within the germplasm involved in the breeding program, since the maximum genetic gain relies on trait heritability and on the deviation – in terms of traits value – of selected individuals from the population mean^22^. Attempts to account for specific germplasm within ideotyping studies have been conducted on different crops, including rice^3,12^, soybean^5^, peach^10^, sunflower and apple^8^. Nevertheless, this approach is feasible only when crop modellers are directly involved in the breeding program and technologies for effectively measuring simple traits corresponding to model parameters are available (e.g.^23^). In any case, deriving distributions by measuring parameter values on specific germplasm requires additional resources compared to deriving generic distributions for a crop from literature values. It might also increase the burden of phenotyping, given model parameters mostly refer to simple traits, which are often either ignored within standard screening activities or estimated in a way that is not consistent with how the trait is formalized in the modelling approach. For instance, breeders often score leaf angle to select for improved canopy architecture^24^, whereas mathematical models include quantitative parameters for describing, e.g., leaf size, shape, curvature, or synthetic parameters (light extinction coefficient) representing the tendency of a canopy (or of a canopy layer) to intercept radiation given a certain leaf area^23^.

Recently, Picheny *et al*.^8^ provided a proof of concept for the need of accounting for actual trait variability to derive feasible ideotypes. However, although tailoring parameter distributions to specific germplasm should theoretically increase the *in vivo* realizability of ideotypes, the actual impact on ideotype profiles has never been quantified. Paleari & Confalonieri^25^ used jackknife resampling to show how uncertainty in parameters distributions can affect sensitivity analysis results. Although this could apply also to ideotyping in theory, the extent of the impact of uncertainty propagation on ideotype profiles cannot be estimated based on Paleari & Confalonieri^25^ results, given they derived alternative distributions using mathematical techniques instead of different sets of observations. Moreover, they observed that the impact of uncertainty in distributions varied greatly according to the parameter considered, with larger effects for parameters driving non-linear functions.

The objective of this study was to evaluate the impact of using parameter distributions defined for a specific germplasm on ideotyping results. Using rice in Northern Italy as a case study, we compared ideotypes based on germplasm-specific distributions with those achieved for the same site by Paleari *et al*.^4^ using generic distributions for rice derived from literature.

## Results

### Statistical distributions of traits

**Figure 1:**
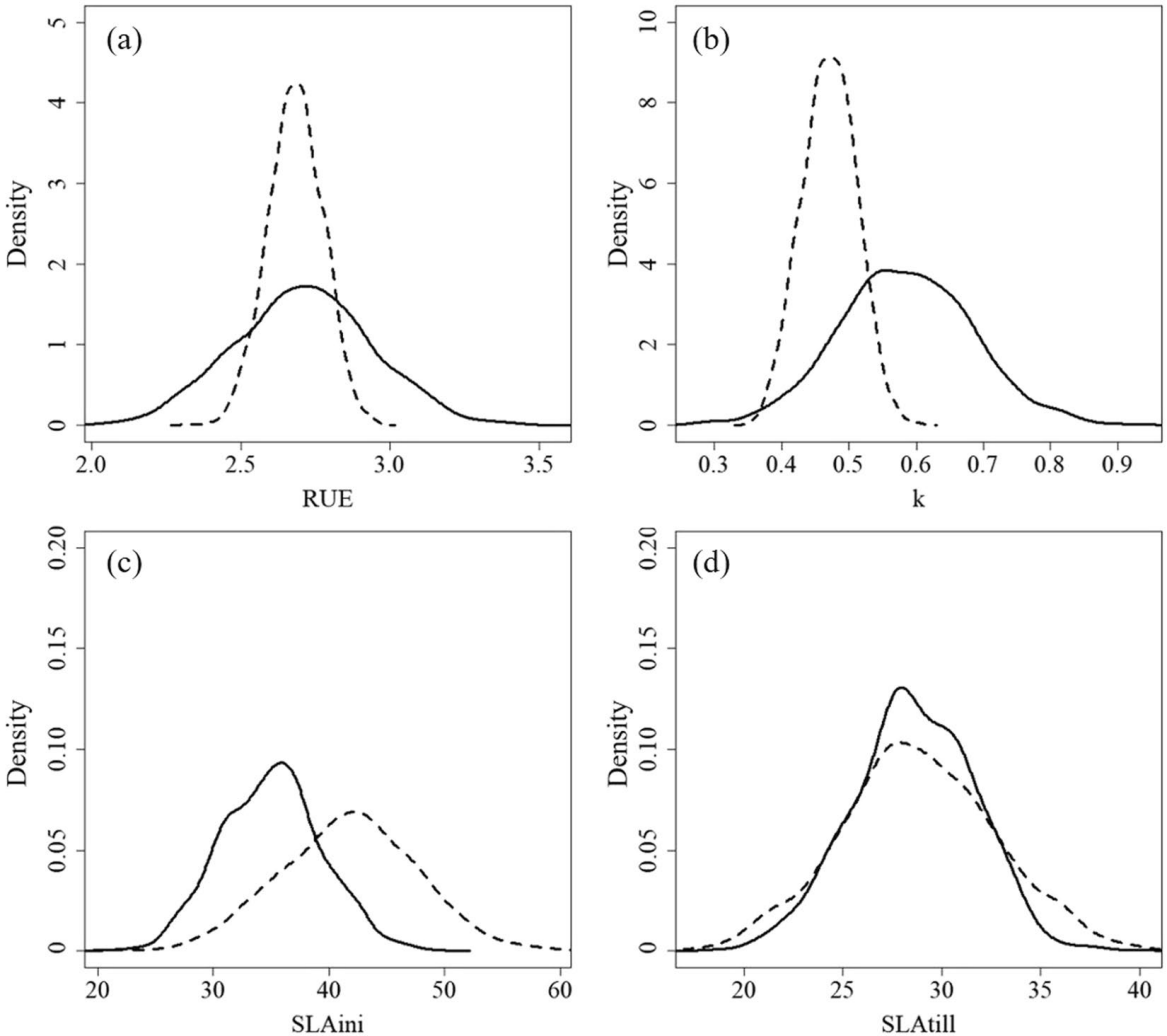
Comparison between germplasm-specific distributions (solid line) and those derived from literature (dotted line) by Paleari *et al*.^4^ for parameters (**a**) radiation use efficiency (RUE, g MJ^−1^), (**b**) canopy light extinction coefficient (k, −), **(c**) specific leaf area at emergence (SLAini, m^2^ kg^−1^) and (**d**) specific leaf area at tillering (SLAtill, m^2^ kg^−1^).

In line with the Paleari *et al*.^4^ study, all the traits analysed were normally distributed according to the Shapiro and Wilk^26^ test, although the analysis revealed marked differences in means and standard deviations between germplasm-specific and literature-derived distributions (Fig. 1). For radiation use efficiency (RUE, g MJ^−1^, Table 1), the two distributions had similar mean (2.68 vs 2.72 g MJ^−1^) but the standard deviation was larger for the values estimated during the field experiment (0.23 instead of 0.09 g MJ^−1^). Differences involved both mean and standard deviation for light extinction coefficient (k, unitless, Table 1), for which the germplasm-specific distribution had higher values for both metrics, i.e., 0.58 vs 0.47 for the mean and 0.1 vs 0.04 for standard deviation.

**Table 1:** Parameters used in the ideotyping study, with acronyms, units, function, distribution derived from literature and, when available, distribution from a dedicated field phenotyping experiment involving Italian rice varieties.

Parameter	Symbol	Process	Literature-derived distribution^a^	Germplasm-specific distribution^b^
Radiation use efficiency	RUE (g MJ^−1^)	Photosynthesis	Normal (*m* 2.68; *s* 0.09)	Normal (*m* 2.72; *s* 0.23)
Light extinction coefficient	k (−)	Canopy light interception	Normal (*m* 0.47; *s* 0.04)	Normal (*m* 0.58; *s* 0.1)
Specific leaf area at emergence	SLAini (m^2^ kg^−1^)	Canopy development	Normal (*m* 41.6; *s* 5.9)	Normal (*m* 35; *s* 4.36)
Specific leaf area at tillering	SLAtill (m^2^ kg^−1^)	Canopy development	Normal (*m* 28.68; *s* 3.89)	Normal (*m* 28.70; *s* 3.18)
Resistance to blast disease	BlastRes (−)	Response to biotic stress	Discrete (1, 2, 3)	
Threshold temperature for chalkiness	T-Chalkiness (°C)	Grain quality	Normal (*m* 26.4; *s* 0.9)	
Threshold temperature for grain breakage	T-HeadRice (°C)	Grain quality	Normal (*m* 23.9; *s* 2.1)	
Threshold temperature for cold-induced spikelet sterility	T-ColdSter (°C)	Response to abiotic stress	Normal (*m* 13.5; *s* 1.4)	
Threshold temperature for heat-induced spikelet sterility	T-HeatSter (°C)	Response to abiotic stress	Normal (*m* 34.4; *s* 1.5)	

In case of normal distribution, m is the mean and s is the standard deviation.

^a^From Paleari *et al*.^4^.

^b^Derived via a dedicated field experiment involving Italian rice varieties (see Table 2 and the Methods section).

Considering specific leaf area (SLA, m^2^ kg^−1^, Table 1), values measured during the field experiment at emergence (SLAini) were largely similar to those retrieved from literature, although a certain tendency towards lower values was found. Mean and standard deviation derived from literature were indeed equal to 41.60 m^2^ kg^−1^ and 5.90 m^2^ kg^−1^, whereas corresponding values for the germplasm analysed were 35.00 m^2^ kg^−1^ and 4.36 m^2^ kg^−1^. At tillering (SLAtill), instead, the two distributions basically overlapped, with 28.70 m^2^ kg^−1^ (mean) and 3.18 m^2^ kg^−1^ (standard deviation) for the germplasm-specific distribution vs 28.68 m^2^ kg^−1^ and 3.89 m^2^ kg^−1^. The similarity of the two distributions for SLAtill was confirmed by the Kolmogorov-Smirnov test (*p*-value <0.05). No correlations were found between the trait values determined during the field experiment, with the highest Pearson’ correlation coefficient equal to 0.13 for RUE and k (not significant). Therefore, corresponding parameters were considered as independent during the sensitivity analysis.

### Implications for ideotype design

**Figure 2:**
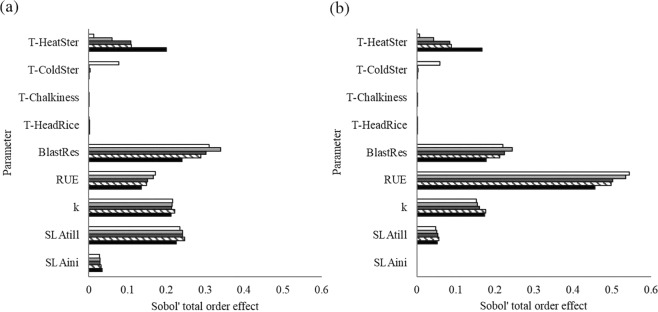
Results of the sensitivity analysis performed with the variance-based method of Sobol’^27^ using literature-derived (**a**) and germplasm-specific (**b**) parameter distributions. Results for both current conditions (baseline, white bars) and climate change scenarios centred in 2030 are reported (light and dark grey bars: RCP2.6-GISS-ES and RCP8.5-GISS-ES, respectively; striped and black bars: RCP2.6-HadGEM2 and RCP8.5-HadGEM2, respectively). T-HeatSter, T-ColdSter, T-Chalkiness and T-HeadRice (°C) are threshold temperatures for heat- and cold-induced spikelet sterility, and for chalkiness and grain breakage, respectively. BlastRes (1 to 3, unitless) is resistance to blast, RUE (g MJ^−1^) is radiation use efficiency, k (unitless) is extinction coefficient for solar radiation, and SLAini and SLAtill (m^2^ kg^−1^) are specific leaf area at emergence and at tillering.

Differences in distributions markedly affected sensitivity analysis results, causing a re-ranking of most relevant parameters according to the total order sensitivity index derived with the Sobol’ method^27^ (Fig. 2). Using germplasm-specific distributions, RUE became the parameter with the highest impact on model output instead of being ranked fourth, whereas the parameter representing resistance to rice blast disease (BlastRes) shifted from the first position to the second. Despite the marked differences between the two distributions for k, the parameter remained the third in the ranking. This confirmed the non-linear response of sensitivity analysis results to perturbation in factor distributions, as reported by Paleari & Confalonieri^25^. The relevance of SLAini and SLAtill was decidedly lower with respect to what achieved using literature-based distributions, especially for SLA at tillering. The top-down concordance coefficient (TDCC^28^) values quantitatively confirmed these considerations, highlighting low concordance between the rankings obtained using the two sets of distributions (average TDCC over different climate scenarios was 0.75). Within parameter, instead, the use of different distributions did not alter the relative response to climate scenarios (e.g., the highest Sobol’ index for RUE was achieved for the baseline and lowest for RCP8.5-HadGEM2 regardless of the distributions).

Figure 2 shows only results for the closest timeframe (2030), since no relevant differences emerged for the long-term scenarios (2050).

**Figure 3:**
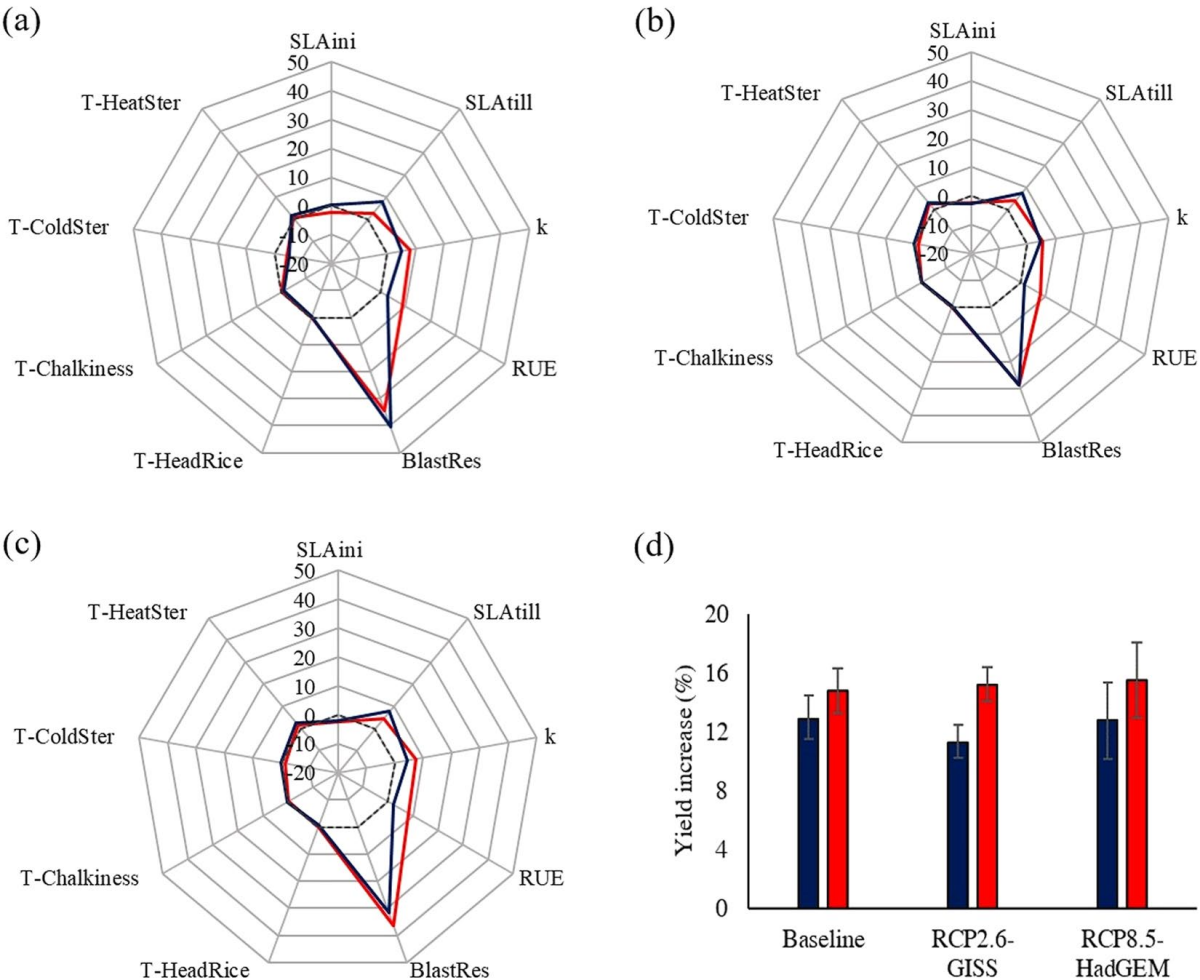
Comparison between rice ideotype profiles derived by using germplasm-specific (red) and literature-derived (blue) parameter distributions for current conditions (**a**) and climate change scenarios centred on 2030, RCP2.6-GISS-ES (**b**) and RCP8.5-HadGEM2 (**c**). Ideotype profiles are presented as percentage variation of parameter values (representing plant traits) as compared to the distribution means (represented by the dotted line). Yield benefits achievable with proposed ideotypes as compared to the genotype defined by the parameter distribution means are also shown (**d**). Error bars refer to the standard deviation of yield benefits over the 20-year timeframe. T-HeatSter, T-ColdSter, T-Chalkiness and T-HeadRice (°C) are threshold temperatures for heat- and cold-induced spikelet sterility, and for chalkiness and grain breakage, respectively. BlastRes (1 to 3, unitless) is resistance to blast, RUE (g MJ^−1^) is radiation use efficiency, k (unitless) is extinction coefficient for solar radiation, and SLAini and SLAtill (m^2^ kg^−1^) are specific leaf area at emergence and at tillering.

Uncertainty in parameter distributions propagated up to ideotype profiles (Fig. 3), although to a lesser extent compared to sensitivity analysis results. Main differences in ideotype profiles were achieved for the parameters SLAtill and RUE, for which the largest variations in sensitivity analysis results were found when the new distributions were used (Fig. 2). The lower relevance obtained for SLAtill when germplasm-specific distributions were used led to reduce the extent of the improvement suggested for this trait (from 8% to 4% by averaging results from all climate scenarios), compared to what achieved using literature-based distributions. Clear changes were found also for RUE, for which germplasm-specific distributions reflected in ideotypes characterized by a suggested increase in photosynthetic efficiency four times higher (+8%, on average, instead of 2%) compared to what achieved by Paleari *et al*.^4^. Smaller variations in the new ideotypes were instead detected for blast resistance – that remained the most important trait – and k. For the latter, the higher variability available in the germplasm analysed turned into wider possibilities of improvement, although the suggested change in the trait value was only slightly affected (+7% instead of +5% by averaging results from all scenarios). Despite a general relationship could be observed between the extent of the differences in distributions and the resulting change in the suggested trait value, results underlined how the effect was clearer for parameters high-ranked by the sensitivity analysis (e.g., RUE).

Differences in ideotype profiles due to alternative parameter distributions affected also their performances (Fig. 3d). Ideotypes defined by using parameter distributions derived from the specific germplasm showed indeed slightly higher yield benefits compared to those defined by Paleari *et al*.^4^, to an extent that ranged between 2 ± 1.5% (baseline) and 4 ± 0.6% (RCP2.6-GISS-ES).

## Discussion

Discrepancies between germplasm-specific and literature-derived distributions can be ascribed to different factors, depending on the specific trait. Concerning RUE, the higher variability observed for the germplasm analysed is in agreement with Boschetti *et al*.^29^, who highlighted large differences in RUE between Italian japonica and tropical japonica varieties, and between old and modern varieties. According to the authors, the reason was related with differences in both physiological and morphological features, especially concerning canopy structure. Improved canopy architecture allows indeed deep penetration of light towards lower leaves and prevent saturation of photosynthetic chains in upper canopy layers^30^, thus maximizing RUE of the whole canopy^31^. In the field experiment carried out during this study, a large heterogeneity in k was indeed observed, leading to a standard deviation more than double the one derived from literature. This could be due either to the wide range of architectural types involved in our study (Table 2) or to the method used for trait estimate. We derived indeed k according to the Campbell ellipsoidal distribution^32^ using direct measurements of leaf angles, whereas it is usually retrieved using indirect methods (e.g.^29,31^). Although some authors found out good correlations between canopy features estimated using direct and indirect methods^33^, this aspect could need further investigations.

**Table 2:** Pool of Italian rice varieties used in the study. Information provided by seed companies.

Variety	Profile	Registration year	Mean cycle length (days)
‘Aiace’	Japonica	2002	130
‘Arborio’	Japonica	1967	155
‘Augusto’	Japonica	2002	140
‘Baldo’	Japonica	1977	150
‘Balilla’	Japonica	1967	155
‘Barone CL^a^’	Japonica	2014	145
‘Brio’	Japonica	2005	145
‘Cammeo’	Japonica	2015	140
‘Caravaggio’	Japonica	2015	150
‘Carnaroli’	Japonica	1983	160
‘Carnise’	Japonica	2008	150
‘Carnise precoce’	Japonica	2008	130
‘Centauro’	Japonica	2002	140
‘Cerere’	Japonica	2009	135
‘Cleopatra’	Japonica	2015	140
‘CRLB1’	Tropical-japonica	2007	120
‘Crono’	Japonica	2010	135
‘Dardo’	Japonica	2010	135
‘Ellebi’	Tropical-japonica	2007	145
‘Fedra’	Japonica	2012	135
‘Galileo’	Japonica	2002	135
‘Generale’	Japonica	2014	150
‘Gladio’	Tropical-japonica	1998	130
‘Gloria’	Japonica	2010	135
‘Karnak’	Japonica	2002	150
‘Keope’	Japonica	2015	140
‘Leonardo’	Japonica	2017	140
‘Loto’	Japonica	1988	130
‘Luna CL^a^’	Japonica	2011	135
‘Mare CL^a^’	Tropical-japonica	2012	140
‘Meco’	Japonica	2012	145
‘Mirko’	Tropical-japonica	2014	140
‘Onice’	Japonica	2011	135
‘Opale’	Japonica	2008	145
‘Puma’	Japonica	2011	140
‘Ronaldo’	Japonica	2010	150
‘Selenio’	Japonica	1987	145
‘Sirio CL^a^’	Tropical-japonica	2009	140
‘Sole CL^a^’	Japonica	2012	140
‘Thaibonnet’	Tropical-japonica	1992	165
‘Ulisse’	Japonica	2007	155
‘Vasco’	Japonica	2013	145
‘Volano’	Japonica	1972	155

^a^Clearfield® technology (resistance to imidazolinone herbicides).

Despite germplasm-specific distributions were derived using data from a single field experiment, management practices (preventing water and nutrient stresses and damages due to biotic factors) and methods for determining trait values provide guarantees on their representativeness. Moreover, in the case of SLAini and SLAtill, the corresponding trait is considered as a stable one (e.g.^34^). Regarding k, it is known to be affected by plant density in different crop species^35^. However, we used in the experiment the plant density suggested by the seed companies that released the varieties; this allows considering the k values we measured as representative. RUE is instead a variable known to be strongly affected by G × E interaction^3^. Nonetheless, under unlimiting conditions for water, nutrients, pests and diseases, the main environmental factor affecting trait value is temperature^31^, which was handled in this study as shown in Eq. 1.

In a previous research aimed at designing rice ideotypes^4^ we underlined the potential limitations of considering a generic, virtual rice germplasm when defining trait distributions. Picheny *et al*.^8^ provided a proof of concept of the need of accounting for real variability in traits to derive feasible ideotypes. However, the real impact of uncertainty in distributions on ideotype features was never quantified. Since breeding programs usually focus on a selected pool of genotypes, we repeated the Paleari *et al*.^4^ study by exploring the actual variability available in a specific germplasm. Despite distributions markedly differed only for two parameters out of four, results showed that the impact on ideotype profiles can be relevant. This highlights the need of increasing the awareness on the consequences of uncertainty in trait distributions within ideotyping studies, whether the objective is suggesting priorities for breeding or screening parameters to include in a successive optimization phase.

## Methods

### Parameters involved in the study

As in Paleari *et al*.^4^, the ideotyping study was limited to model parameters with a close link to plant traits on which rice breeders are focusing on (Table 1). Among them, germplasm-specific distributions were derived via field phenotyping only for parameters involved with canopy structure (i.e., specific leaf area at emergence and tillering [SLAini and SLAtill, respectively, m^2^ kg^−1^] and light extinction coefficient [k, unitless]) and with photosynthetic efficiency (radiation use efficiency [RUE, g MJ^−1^]). For the other parameters we used literature-derived distributions (Table 1). Given that the objective of this research was indeed to provide a proof of concept of the relevance of using germplasm-specific parameter distributions, involving traits of resistance/tolerance to biotic and abiotic stressors would have required extensive trials under controlled conditions (e.g., generating conditions favourable to pathogens or extreme weather events), which was beyond the scope of this study.

Both germplasm-specific and literature-derived distributions were defined by using only data collected under potential growing conditions (no limiting factors other than radiation and temperature). This was decided to avoid including in parameter values factors involved with environmental variables or management practices, which would have compromised the capability of the model to explicitly reproduce G × E × M interactions.

The Italian rice varieties used to define germplasm-specific distributions are presented in Table 2, whereas literature-derived distributions were defined by using values measured on rice varieties from all over the world, without targeting specific ecotypes or subpopulations. They include indica, japonica and tropical-japonica type varieties from Asia (e.g., China, Philippines, Indonesia, Japan), North and South America (e.g., USA and Brazil), Europe (e.g., Italy and Spain), Africa (e.g., Senegal), and Australia. Both traditional and high-yielding varieties were included. More details on the genotypes involved can be found in Paleari *et al*.^4^.

### Field experiment and definition of statistical distributions for traits

To derive trait distributions representative of the germplasm available for rice breeding in Italy, 43 varieties were selected based on their relevance as perceived by breeders and seed companies and on harvested area in the last three years (source: Italian National Rice Authority), the latter indicative of the appreciation of market and farmers. For each variety, RUE, k, SLAini, and SLAtill were measured during a dedicated field experiment (Table 1). Traits related with plant height and phenological development were also collected to further characterize the varieties. The representativeness of the selected pool of varieties (Table 2) is demonstrated by the heterogeneity in morphological and physiological features, which reflects different market and breeding targets^36^.

This is clear from the variability in, e.g., mean harvest index (from about 0.3 [‘Carnaroli’] to 0.6 [‘Loto’]) and cycle length (120 [‘CRLB1’] to 165 [‘Thaibonnet’] days), registration year (from 1967 [‘Arborio’] to 2017 [‘Leonardo’]), canopy structure (from horizontal [‘Gloria’] to erect [‘Dardo’] leaves), height (from 50 cm [‘Fedra’] to almost 120 cm [‘Carnaroli’]). Most varieties are japonica, well adapted to temperate areas, although the introduction of American and Asian germplasm has led to tropical-japonica profiles with indica-like features^37^.

Rice varieties were row seeded on May 18, 2017 (mean density = 200 plants m^−2^) in 30 m^2^ (10 m × 3 m) plots and flooded from the 4th leaf stage until maturity. Plots were maintained under optimal conditions for nutrients (135 kg N ha^−1^ applied as urea) and water (continuous flooding), and treatments allowed keeping them weed-, pest- and disease-free. Temperatures recorded during the 2017 rice season were in line with the average conditions in the site (Gaggiano, Northern Italy, 45.24 N, 9.02 E, 117 m a.s.l.), the only exception being the slightly higher temperatures recorded in June and August, which in any case did not exceed optimal values for rice (monthly average of daily mean temperature equal to 24 °C and 27 °C in June and July, respectively). Precipitations during the 2017 season were lower than the long-term average, especially during summer months. However, this did not affect the crop because of the flooding conditions.

SLAini (BBCH 11) and SLAtill (BBCH 29) were determined on three plants per plots by scanning all the leaves of the sampled plants and then drying them until constant weight. The values of k for each variety were estimated at late heading (BBCH 58) with the PocketPlant3D smartphone application^23^, which uses the device accelerometer and magnetometer to measures all leaf angles from the insertion to the tip and then derives k based on the Campbell ellipsoidal distribution^32^. Even in this case, the sample size was three plants per plot.

RUE was derived according to Boschetti *et al*.^29^ (Eq. 1):

1 $$RUE=\frac{AG{B}_{\Delta t}}{0.5\cdot RA{D}_{\Delta t}\cdot (1-{e}^{-k\cdot LA{I}_{\Delta t}})}\cdot \frac{1}{Tli{m}_{\Delta t}}$$

where *AGB*_*Δt*_ is the aboveground biomass (AGB, t ha^−1^) accumulated in the time interval *Δt*; *RAD*_*Δt*_ is the cumulated global solar radiation in *Δt*; 0.5 is a factor to convert global solar radiation in photosynthetically active radiation (*PAR*); *k* is the light extinction coefficient estimated for each variety as described above; *LAI*_*Δt*_ and *Tlim*_*Δt*_ are, respectively, the mean leaf area index and the mean thermal limitation during *Δt*. Thermal limitation was estimated as a function of mean daily air temperature, with cardinal temperatures (minimum, optimum and maximum) equal to 12 °C, 28 °C and 38 °C. To limit the impact of uncertainties in the estimation of intercepted PAR before the close canopy stage and of the effects of senescence on RUE, *Δt* was considered as the interval between panicle initiation (BBCH 30) and late heading (BBCH 58). At these phenological stages, leaf area index was estimated using the smart app PocketLAI^38^ as the average of five replicates per plot, and aboveground biomass was determined on 20 plants per plot.

Once SLAini, SLAtill, k, and RUE values were estimated for each of the 43 varieties, statistical distributions were derived and compared with those used by Paleari *et al*.^4^ using the Kolmogorov-Smirnov test.

### Crop model and ideotype design

As in Paleari *et al*.^4^, the hourly version of the rice model WARM^39^ was used. Biomass accumulation is reproduced using a net photosynthesis approach, with RUE modulated by temperature stress, saturation of enzymatic chains, senescence, diseases and atmospheric CO_2_ concentration. A micrometeorological module allows using mid-canopy temperature for temperature limitation to photosynthesis and temperature at the meristematic apex for phenological development and spikelet sterility. Daily accumulated AGB is allocated to the different plant organs using a set of beta and parabolic functions driven by development stage (DS), with the biomass partitioned to leaves converted in leaf area using a DS-dependent specific leaf area. Given LAI and k, Lambert-Beer law is used to estimate the fraction of radiation intercepted by the canopy^40^. The interaction between rice and airborne fungal pathogens along the different phases of the epidemic is simulated as function of host resistance, air temperature, humidity and leaf wetness, by using a generic disease model that can be adapted to specific pathosystems via different parameter sets^41^. In particular, the impact of blast disease (causal agent: *Magnaporthe oryzae B.Couch*) on crop growth is reproduced through the reduction of photosynthetic tissues (sporulating lesions), radiation use efficiency and photosynthates partitioning to panicles; this allows simulating both leaf and neck blast^41^. Spikelet sterility because of cold (pre-flowering) and heat (flowering) stress is estimated as a function of variety-specific temperature thresholds, modulated according to bell-shape factors to account for heterogeneity in culm development. Chalkiness and head rice yield are simulated, respectively, based on growing degrees daily accumulated above a critical temperature after heading and on nighttime temperature, wind speed, rainfall and temperature stress during grain filling^42^.

Among the five sites of the Paleari *et al*.^4^ study, we focused here on the Italian one because of the specific germplasm analysed, and we used the same weather data and ideotyping methodology to allow a clear quantification of the differences due to the distributions adopted. Climate data consisted of 20-year daily weather series for current conditions (baseline, 1986–2005) and future projections centred on 2030 and 2050 to support the identification of breeding targets also in the medium term. Climate change scenarios derived from the combination of two representative concentration pathways (RCP, IPCC 2013^43^) – RCPs 2.6 and 8.5 – and two general circulation models (GCM) – HadGEM2 (Hadley Centre, UK^44^) and GISS-ES (NASA^45^). Downscaling was performed using the Climak weather generator^46,47^. For the ideotyping, parameter hyperspace was explored using the Monte Carlo sampling technique implemented in the Sobol’^27^ global sensitivity analysis method, which was parameterized to 5120 combinations (>500 · number of parameters under analysis)^39^. Germplasm-specific distributions for RUE, k, SLAini and SLAtill were derived from the field experiment, whereas distributions for the other parameters (blast resistance [BlastRes], threshold temperatures for heat- [T-HeatSter] and cold-induced sterility [T-ColdSter], chalkiness [T-Chalkiness] and head rice [T-HeadRice]) were the same used by Paleari *et al*.^4^. The output variable on which sensitivity metrics were calculated was the value produced per hectare to allow accounting for both productivity and grain quality. Parameters ranking was based on the Sobol’ total order sensitivity index, and agreement between the rankings obtained with germplasm-specific and literature-derived distributions was quantified using the top-down concordance coefficient (TDCC^28^). The combinations of parameters (representing potential varieties) were then ranked according to the index proposed by Paleari *et al*.^4^ (Ideotype score, *I*_*score*_, unitless, Eq. 2):

2 $${I}_{score}=[\mathop{\sum }\limits_{i=1}^{n}((\frac{|{x}_{i}-{m}_{i}|}{{m}_{i}}\cdot 100)\cdot \frac{1}{\sqrt{{S}_{i}}})\cdot \frac{1}{n}]\cdot (1-\frac{{Y}_{v}}{{Y}_{vmax}})$$

where *n* is the number of parameters defining the ideotype, *x*_*i*_ is the value of the *i*th parameter, *m*_*i*_ is the distribution mean of the *i*th parameter, *S*_*i*_ is the Sobol’ total order effect for the *i*th parameter, *Y*_*v*_/*Y*_*v max*_ is the production of the ideotype (expressed as € ha^−1^) normalized to the maximum of all ideotypes under evaluation. Ideotype profiles were derived by averaging parameter values of the 1% top-ranked combinations to minimize the risk of identifying ideotypes corresponding to local minima in the parameter space, i.e., to combination of plant trait values surrounded others leading to poor plant performance. This index allowed considering both the performances and the feasibility of potential ideotypes, the latter evaluated in terms of (i) the required extent of trait improvement as compared to the population mean and (ii) the impact of traits variation on yield (via the value of *S*).

## Data Availability

The datasets analysed in the study are available from the corresponding author upon motivated request.
